# Fit for purpose – Selecting the best mitochondrial DNA for the job

**DOI:** 10.1016/j.cmet.2024.06.011

**Published:** 2024-07-02

**Authors:** Sarah J Pickett, Robert W Taylor, Robert McFarland

**Affiliations:** 1 Mitochondrial Research Group, Faculty of Medical Sciences, Newcastle University, Newcastle upon Tyne, NE2 4HH, UK; 2 Biosciences Institute, Faculty of Medical Sciences, Newcastle University, Newcastle upon Tyne, NE2 4HH, UK; 3 Translational and Clinical Research Institute, Faculty of Medical Sciences, Newcastle University, Newcastle upon Tyne, NE2 4HH, UK; 4 NHS Highly Specialised Service for Rare Mitochondrial Disorders, Newcastle upon Tyne Hospitals NHS Foundation Trust, Newcastle upon Tyne, NE1 4LP, UK

Mitochondria probably evolved into their present form following an ancient endosymbiotic fusion event that offered a competitive reproductive advantage to anaerobic hosts. One obvious remnant of that primeval fusion in humans is the persistence of a small (16.5kb) circular, double-stranded molecule of mitochondrial DNA (mtDNA). Present in almost all human cells, with the notable exception of red blood cells, mtDNA encodes 22 tRNAs, 2 rRNAs and 13 proteins crucial for oxidative phosphorylation - a process that allows the cell to meet its essential requirements for adenosine triphosphate (ATP) production. Mitochondria contain multiple copies of mtDNA (10-100 copies per mitochondrion) and cells densely packed with mitochondria, such as oocytes and cardiomyocytes, may contain several hundred thousand molecules of mtDNA. Mutations arising in these independently replicating molecules can therefore result in more than one population of mtDNA or genotype within the same cell - a situation referred to as heteroplasmy.

Most of the genetic variants in mtDNA are of no clinical consequence, but pathogenic mutations are carried at very low heteroplasmy by approximately 1 in 200 individuals^1^ and cause disease at much higher heteroplasmy in 1 in 5000 people^2^. At a cell and tissue level, the inherent redundancy afforded by a multicopy genome permits many pathological mtDNA mutations to be tolerated until a cell and mutation specific threshold is exceeded, this is variably between 60% and 90% heteroplasmy. Above this threshold oxidative phosphorylation is disrupted and cellular production of ATP severely hampered, leading to an energy deficit, attempted metabolic remodelling and cellular dysfunction with initiation of a cell stress response (Figure 1A).

Although pathological mtDNA mutations may arise sporadically, most are maternally inherited and pass through a ‘mitochondrial genetic bottleneck’ during early germline development that, coupled with the passive segregation of mtDNA during cytokinesis, can result in markedly different intergenerational heteroplasmy – an important consideration for genetic counselling given higher heteroplasmy is generally associated with early-onset, severe mitochondrial disease. In post-mitotic tissues, heteroplasmy typically remains stable, though mtDNA deletions of varying sizes and somatic mtDNA mutations have been shown to accumulate with age in post-mitotic cells, contributing to late-onset, common disease pathology. This is often not the situation for mitotic tissues, such as blood, where heteroplasmy for specific mutations including m.3243A>G and single large-scale deletions, has been noted to steadily decline over a time-course of years. In contrast, sporadic mutations of mtDNA in colonic crypt cells can quickly rise to high levels of heteroplasmy over the course of a few cell divisions. At a tissue level, heteroplasmy describes a mean value for the sample assayed and doesn’t reflect the remarkable cell-to-cell variation observed for some mtDNA mutations, such as m.8993T>G in *MT-ATP6*, where adjacent cells can harbour almost undetectable or near homoplasmic mutation loads. Again, an important consideration in offering reproductive options such as preimplantation genetic testing.

The factors determining intracellular steady-state heteroplasmy have remained elusive and lines of evidence from both cultured cell lines and mouse models have been conflicting regarding the processes of random genetic drift or active selection as the prevailing mechanism^3,4^. Recent high throughput single cell analysis in mouse models at different ages has indicated that heteroplasmy variance is the same for rapidly dividing spleen cells and non-dividing neurons^4^.

Kotrys and colleagues utilised mtDNA base editing technology^5^ and a new technique, single cell combinatorial indexing leveraged to interrogate targeted expression (SCI-LITE), to explore the dynamics of intracellular heteroplasmy^6^. SCI methodology employs sequential rounds of split-pool molecular barcoding to identify single cells with a high degree of certainty^7^. SCI-LITE adapts this approach to capture only selected transcripts, rather than bulk RNA, thereby improving scalability and costs. Following validation of this approach in distinguishing between HeLa and 293T human cells and accurately detecting mtRNA levels, the authors combined SCI-LITE and cellular ancestry tracing (a lentiviral based barcoding technique) to allow them to simultaneously measure heteroplasmy and cell lineage (Figure 1B). They engineered cells to harbour either a non-synonymous or synonymous mutation in the *MT-ND4* gene encoding a structural component of mitochondrial complex I (NADH:ubiquinone oxidoreductase), a ~1MDa multimeric assembly of 44 subunits encoded by both the nuclear and mitochondrial genomes^8^. Complex I is a key entry point to the respiratory chain from the TCA cycle, thus coupling cellular and mitochondrial metabolism and complex I dysfunction is the most frequently identified (biochemical) cause of mitochondrial disease pathology^9,10^. The authors showed that while heteroplasmy was generally stable within lineages over time there was a strong negative selection against cells with the non-synonymous *MT-ND4* mutation, while synonymous mutations were maintained, suggesting that cellular fitness is the key driver of selection (Figure 1C&D). The authors next demonstrated that under different circumstances where the *MT-ND4* non-synonymous mutation might confer a cell fitness advantage – severe hypoxia (1% oxygen), Hürthle cell carcinoma lineage, and culture in oligomycin – there was, at the very least, maintenance of non-synonymous mutation heteroplasmy and, in the case of oligomycin treatment, a demonstrable positive selection (Figure 1E).

This detailed, innovative work by Kotrys *et al* offers a fascinating insight into the factors determining heteroplasmy shifts in rapidly dividing immortalised cells *in vitro* and introduces a technology, in the form of SCI-LITE, that will be a powerful tool for further investigation of these factors. Cell culture is a rather artificial environment and it may well be that factors which predominate *in vitro* play a much smaller role *in vivo*. For instance, it is difficult to see how cell fitness alone can explain the slow and steady shift in blood heteroplasmy observed in patients harbouring the m.3243A>G mutation, while similarly pathogenic mtDNA variants, such as m.8344A>G, remain constant over many years and others, including primary LHON variants, are typically homoplasmic in blood throughout life. Is there a mutation specific factor exerting a strong influence on heteroplasmy as suggested by Glynos *et al*.^4^?

Studying post-mitotic cells *in vivo* poses a different set of challenges, but these are arguably the most important cells with regard to disease and ageing. It is likely that the balance of factors determining heteroplasmy within these post-mitotic cells is different again to rapidly dividing cells. In all of these scenarios there are probably multiple factors and processes in play, including random genetic drift and negative selection based on cellular fitness. The predominance of one mechanism over another then depending on the metabolic requirements and environment of the cell, or put more simply, selecting the best mitochondrial DNA for the job.

## Figures and Tables

**Figure 1:**
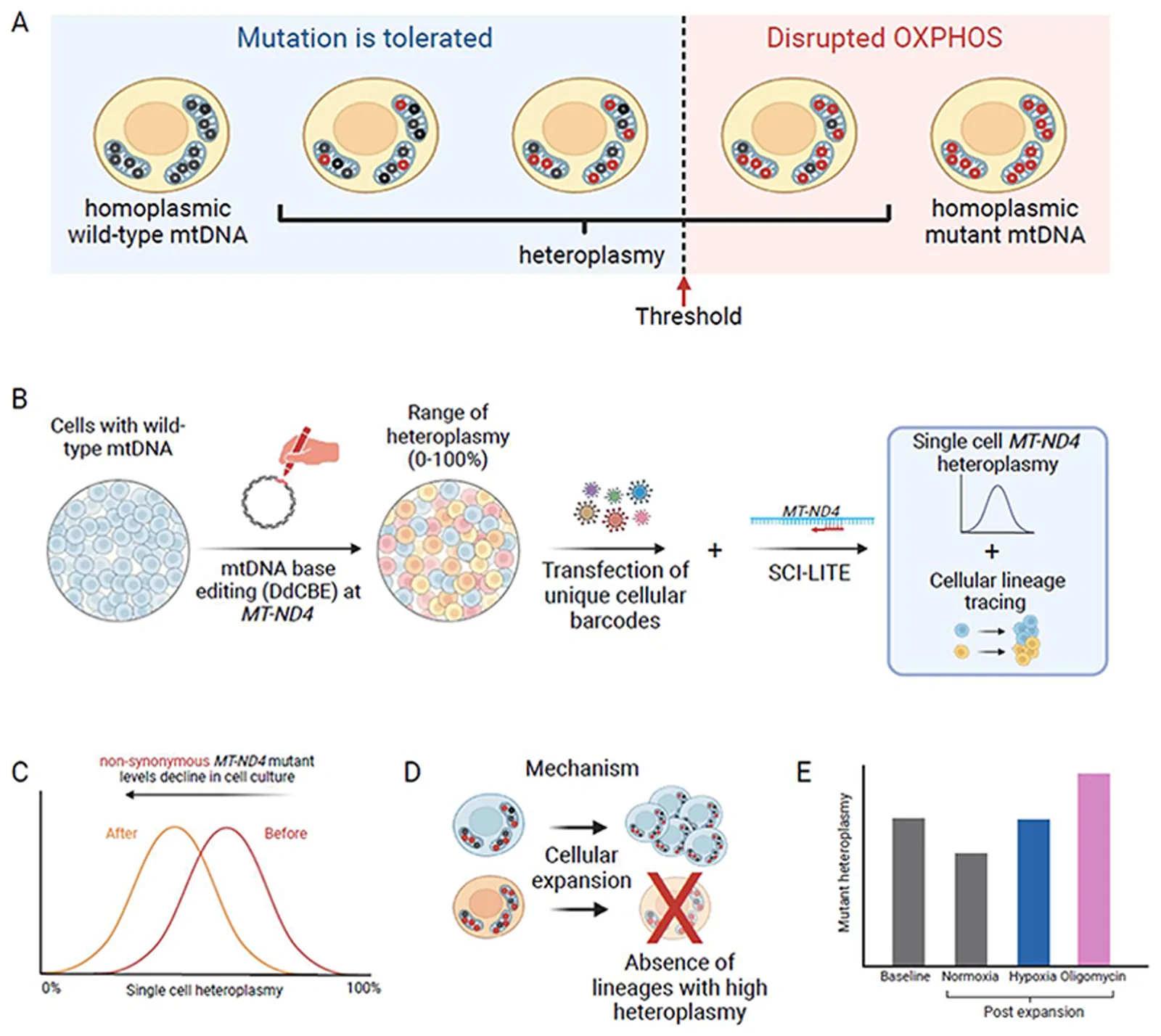
Mitochondrial DNA heteroplasmy and selection. **A**: Both wild-type (black) and mutant (red) mtDNA molecules can co-exist within the same cell. Cells tolerate the presence of mutant molecules until they reach a threshold level, at which point they develop a deficiency in oxidative phosphorylation (OXPHOS). **B**: Schematic representation of single cell genomic techniques employed by Kotrys and colleagues to study the dynamics and mechanisms of mtDNA selection. **C**: In typical cell culture conditions, the non-synonymous MT-ND4 mutation undergoes negative selection. **D**: A key driver of this selection is cell fitness: cell lineages with lower mutant heteroplasmy proliferate whist those with higher mutant levels do not survive. **E**: Cell fitness is determined by the culture environment; mutations are selected against in normoxia (21% O2), heteroplasmy is maintained in hypoxia (1% O2) and mutations are selected for in the presence of oligomycin, a mitochondrial OXPHOS (complex V) inhibitor.
